# Supplementary material for: Quantitative Effects of Temperature and Exposure Duration on the Occurrence and Repair of Indirect Chilling Injury in the Fall Armyworm Spodoptera frugiperda
Source: Insects. 2023 Apr 3;14(4):356. doi: 10.3390/insects14040356 (PMC10145330; doi:10.3390/insects14040356)
Supplement: Supplementary file 1 [file insects-14-00356-s001.zip › insects-2257875-supplementary.pdf]

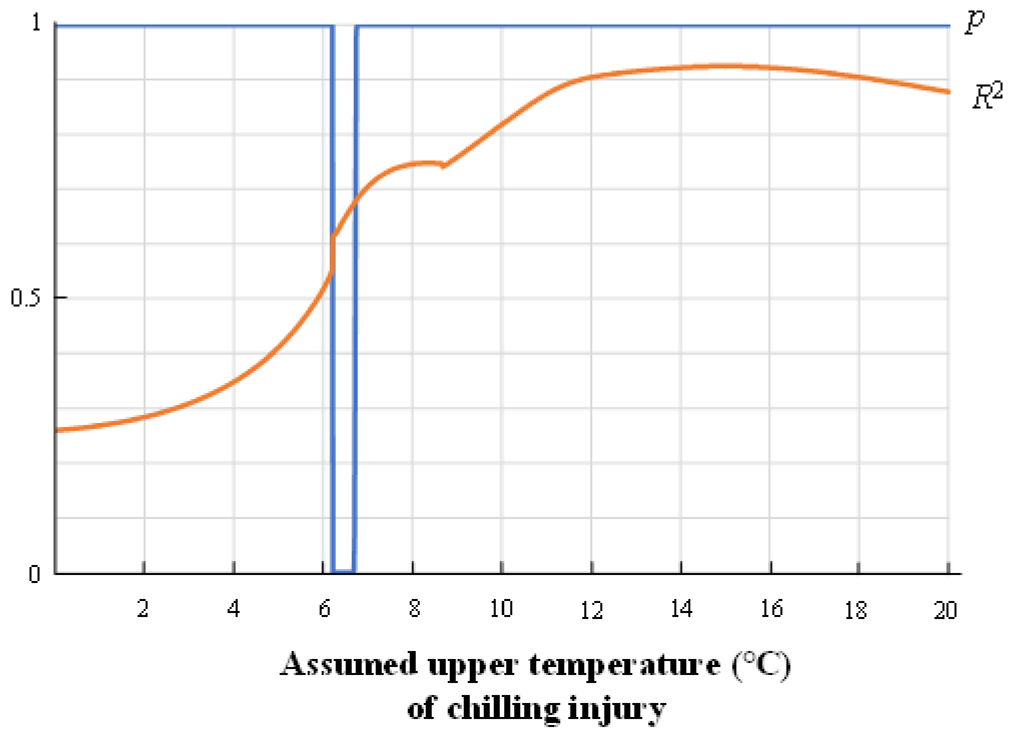

Supplemental Figure S1. Changes in R^2 and P values of a fitted time–temperature model with changes in the assumed upper-limit temperature for chilling injury *c* (Nedvěd et al. 1998; see main text for equation). The best values for parameters *a* and *b* were estimated by the least square method for each value of *c* at intervals of 0.01 °C.
